# Pathobiology of atopic dermatitis and association with disease severity after acute oral steroid treatment

**DOI:** 10.1016/j.jacig.2026.100696

**Published:** 2026-03-28

**Authors:** Emma Price, Christiane Whetstone, Dhuha Al-Sajee, Nadia Alsaji, Karen Howie, Caroline Munoz, Roma Sehmi, Hermenio Lima, Gail M. Gauvreau

**Affiliations:** a Division of Respirology, Department of Medicine, McMaster University, Hamilton, Ontario, Canada; b Firestone Institute for Respiratory Health, Department of Medicine, McMaster University, Hamilton, Ontario, Canada; c Division of Allergy and Immunology, Department of Medicine, McMaster University, Hamilton, Ontario, Canada

**Keywords:** Atopic dermatitis, inflammation, prednisolone, intradermal allergen challenge, skin lesions, cytokines, T_H_2/T_H_1/T_H_17 pathways, EASI, IGA, POEM, SCORAD

## Abstract

**Background:**

Atopic dermatitis (AD) is a chronic inflammatory skin disease with complex immune dysregulation. Acute oral corticosteroids relieve measures of AD severity during flares; however, the association of disease severity with anti-inflammatory effects in skin remains unclear.

**Objective:**

We sought to evaluate the association between clinical severity and skin inflammation in AD.

**Methods:**

Sixteen patients with moderate to severe AD were randomized 1:1 to prednisolone (0.75-0.25 mg/kg tapered over 15 days) or placebo following an 8-day run-in without systemic anti-inflammatory medications. Clinical scores were measured and biopsies from lesional and allergen-challenged skin were collected and analyzed by histology, immunofluorescence microscopy, and ELISA for cells and cytokine levels.

**Results:**

Posttreatment day 8, prednisolone improved clinical scores for Eczema Area and Severity Index (EASI), SCORing Atopic Dermatitis (SCORAD), and Investigator’s Global Assessment (IGA), suppressed eosinophils, basophils, and select T_H_17/proinflammatory cytokines (IL-17A, IL-1β, and TGF-α) in allergen-challenged skin, reduced T_H_2 (IL-5, IL-9, IL-10, and IL-13) and T_H_1 (TNF-α) cytokines, and reduced chemokines (MIP-1β and TARC) in skin lesions (*P* < .05). IL-13, TNF-α, IFN-γ, and MCP-1 levels positively associated with the EASI, SCORAD, and IGA (*r* > 0.5; *P* < .05) in both allergen-challenged and lesional skin.

**Conclusions:**

Prednisolone modulated a broad range of inflammatory pathways in acute versus chronically inflamed AD skin. Furthermore, identification of positive associations between inflammation and clinical outcomes supports the development of therapeutics beyond type 2 inflammation.

Atopic dermatitis (AD) is a chronic, relapsing inflammatory skin disease characterized by dry, itchy, and inflamed skin. AD flares managed with oral corticosteroids provide rapid symptomatic relief and transient control, but this treatment has been associated with adverse effects, including hyperglycemia, mood disturbances, sleep disruption, and gastrointestinal irritation. Prolonged or repeated courses of oral corticosteroids can lead to significant complications such as osteoporosis, hypertension, adrenal suppression, metabolic syndrome, cataracts, and increased susceptibility to infections. Hence, treatments that avoid broad immunosuppression and selectively target active immunologic processes in AD are sought.

The acute phase of AD is primarily mediated by T_H_2-skewed immune responses, triggered by the disruption of the epidermal barrier and injury to keratinocytes. Barrier disruption and keratinocyte injury lead to the release of dermal alarmins (IL-25, IL-33, and TSLP), which activate dendritic cells and type 2 innate lymphoid cells (ILC2s) to promote IL-5 and IL-13 production. This enhances eosinophil recruitment and T_H_2 polarization, exacerbating barrier impairment and sustaining pruritus, creating a self-amplifying inflammatory loop.^1^, ^2^, ^3^, ^4^ IgE further contributes by binding Langerhans cells, dendritic cells, and mast cells, the latter being a major source of IL-4 and IL-13 in AD.^5^^,^^6^ Chronic AD often exhibits signs of evolving immune features, including increased T_H_1 and T_H_17 responses and a broader infiltration of T-cell subtypes, contributing to a complex inflammatory environment.^7^ This heterogeneity may limit the efficacy of single-cytokine–targeting approaches. Understanding of the complex immune milieu is needed to develop novel treatments targeting additional immune pathways, such as T_H_1, T_H_17, T_H_22, and mast cell–mediated inflammation.

Although blockade of IL-4/IL-13 with dupilumab significantly improves measures of AD disease severity, seemingly through a reduction of chronic lesions, pruritus, and IgE levels,^8^, ^9^, ^10^, ^11^, ^12^ treatment with biologics blocking eosinophils,^13^^,^^14^ IgE,^15^^,^^16^ and alarmin cytokines TSLP and IL-33^17^^,^^18^ has not demonstrated therapeutic benefit, suggesting that these inflammatory pathways are not as crucial for the maintenance of chronic disease. To expand our understanding of positive drug targets for treatment of AD, we quantified the effect of acute dosing with oral corticosteroids on inflammatory immune cells and cytokines in chronic lesions of patients with AD and after intradermal allergen challenge (IDAC) to examine their association with improved disease control.

## Methods

### Patient eligibility

Patients between the ages 18 and 65 years with moderate to severe AD, as determined by the Eczema Area and Severity Index (EASI) score at screening, and the development of a positive late cutaneous response to IDAC were recruited. Patients withheld treatment with immunosuppressive or immunomodulating drugs for 4 weeks, treatment with an investigational therapy and biologics for 8 weeks or 5 half-lives, and the initiation of prescription moisturizers. Patients discontinued the use of any antihistamines or doxepin 5 days before study baseline measurements (day 0) and did not change the use of prescription moisturizers during the study. The Hamilton Integrated Research Ethics Committee approved the study, and all patients provided written informed consent. CONSORT reporting guidelines were used.

### Study design

**Fig 1:**
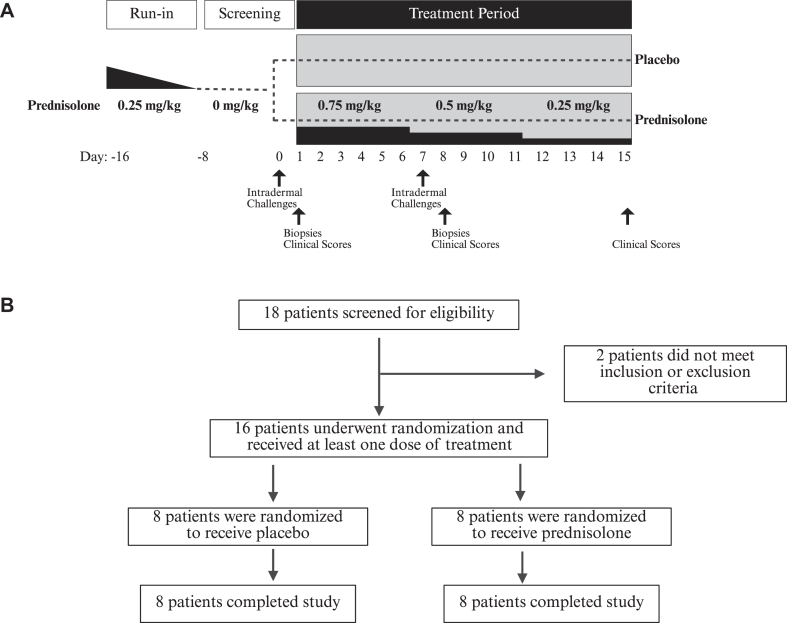
**A,** Study design. All patients completed a run-in period of prednisolone. Patients were then given a low dose of oral prednisolone (0.25 mg/kg) for 8 days and they discontinued the use of prednisolone thereafter. Patients were then randomized 1:1 to receive either prednisolone or placebo for 15 days. Prednisolone dosage was tapered using the following regimen: 5 days at 0.75 mg/kg, 5 days at 0.5 mg/kg, and 5 days at 0.25 mg/kg. Intradermal challenges were conducted on days 0 and 7, and blood and skin samples were collected 24 hours after challenges on days 1 and 8. **B,** CONSORT diagram summarizing the flow of patients through randomization, treatment, and follow-up.

Sixteen days before the start of the study, patients underwent a run-in period consisting of low-dose oral prednisolone (0.25 mg/kg) for 8 days and discontinued use of prednisolone thereafter. Screening tests at day 0 included skin prick test and IDAC. Those developing a late cutaneous response 24 hours after IDAC were enrolled into the study on day 1 and underwent pretreatment clinical skin evaluations consisting of EASI, SCORAD (SCORing Atopic Dermatitis), IGA (Investigator’s Global Assessment), DLQI (Dermatology Life Quality Index), and POEM (Patient-Oriented Eczema Measure) scores and collection of punch biopsies. Patients were then randomized 1:1 using a sequentially numbered computer-generated allocation sequence to begin a 15-day double-blind treatment period of either placebo or prednisolone tapered using the following regimen: 5 days at 0.75 mg/kg, 5 days at 0.5 mg/kg, and 5 days at 0.25 mg/kg. After 7 days of treatment, the skin prick test and IDAC were repeated. Punch biopsies were obtained at day 8 and clinical skin evaluations were conducted at days 8 and 15 posttreatment (Fig 1).

### Skin prick test

A skin prick test using a standard panel of 14 aeroallergen extracts, as well as a positive control (1 mg/mL histamine) and negative control (0.9% saline), was applied to the lower back by pricking the skin. The allergen that produced the largest wheal and demonstrated significant IgE levels by RAST, and confirmed to elicit clinical symptoms was selected and then titrated to a dilution that resulted in a wheal size of 3 × 3 mm (length × width).

### IDAC and skin sampling

The selected allergen dilution (100 μL) was injected intradermally in 2 adjacent and standardized locations on the patient’s back, beside a 100-μL diluent (0.9% saline) control. The site chosen for intradermal challenge was on the patient’s lower back and did not include inflamed skin with ongoing visible AD. The wheal size of the early cutaneous response and late cutaneous response was measured at 10 minutes and 24 hours, respectively, after challenge. At 24 hours after each IDAC when maximum infiltration of immune cells occurs^19^, ^20^, ^21^, ^22^ (corresponding with study days 1 and 8), a punch biopsy was taken from the center of each site using a sterile 4-mm skin punch. At the same time points, an additional punch biopsy was taken from the center of an active lesion and from a location of unaffected skin on the patient’s back.

### Immunofluorescence staining and microscopy

Skin biopsies were formalin-fixed and embedded in paraffin blocks and cells were quantified using standard methods.^23^ Tissue sections were stained by hematoxylin and eosin (H&E) and by indirect immunofluorescence microscopy for activated eosinophil cationic protein (EG2 antibody), major basic protein (MBP), IL-5Rα (CD125), eosinophil progenitor (EoP) cells (defined as CD34^+^CD125^+^ Von Willebrand factor^−^), basogranulin (2D7 antibody), mast cells (tryptase), and IL-33. Histologic determination of dermal lymphocytic and neutrophilic infiltration density by H&E staining was semiquantitatively graded using a previously described 4-point scale^24^: 0, absent; 1, minimal; 2, moderate; and 3, severe. Epidermal thickness was calculated as the total area of the epidermis (mm^2^) divided by length (mm).^25^

### Analysis of cytokines

Skin biopsies were also minced in PBS using a method provided by Eve Technologies to generate a fluid phase from tissue for measurement of inflammatory mediators using a 65-plex Discovery assay (Human Cytokine Array/Chemokine Array 65-Plex Panel; catalog no. HD65; Eve Technologies, Calgary, Alberta, Canada) based on the Luminex technology and Millipore assay targeting 65 specific cytokines.^26^, ^27^, ^28^ Analyte concentrations were extrapolated from a standard curve. Only 56 of the 65 cytokines were detectable in the samples. Missing samples were assigned the group mean, and samples with analyte concentrations less than the lower limit of detection were assigned 50% of the lower limit of detection.

### Statistical analysis

The study was powered using data from a previous study^29^ to detect 50% attenuation in late cutaneous response between prednisolone and placebo. Data were included from all patients who completed up to day 65 per protocol. The Wilcoxon matched-pairs test was used to compare measures of inflammation in lesional and allergen-challenged skin. The Mann-Whitney *U* test was used to compare the effect of prednisolone and placebo on the percent change from pretreatment measurements of clinical outcomes, early and late cutaneous responses, and levels of inflammatory cells and cytokines measured in skin by microscopy and ELISA. Pearson multiple linear regression analyses were performed to test the association of the clinical scores with the inflammatory cells and cytokines measured from the allergen-challenged and lesional skin biopsies. For detailed clinical and laboratory methods, see this article’s Methods section in the Online Repository at www.jaci-global.org.

## Results

### Patient demographic and clinical characteristics

Sixteen patients aged 18 to 65 years were randomized, and the patients completed the study without study-related adverse events or serious adverse events. At day 0 before the start of the treatment period, 2 patients had mild AD, 6 had moderate AD, and 8 had severe AD as determined by the EASI score. Patient demographic characteristics were similar in the groups, with no statistically significant differences between the placebo and prednisolone before the start of treatment (Table I). House dust mite was used for IDAC in 15 of the 16 patients.

**Table I:** Baseline demographic and clinical characteristics of patients

Characteristics	Total (N = 16)	Placebo (n = 8)	Prednisolone (n = 8)
Race			
White	14	6	8
Asian	1	1	0
East Indian	1	1	0
Age (y)	37.3 ± 15.7	34.8 ± 16.9	39.8 ± 15.2
Sex			
Male	10	5	5
Female	6	3	3
Height (cm)	173.3 ± 12.2	173 ± 11.1	173.7 ± 14.24
Weight (kg)	79.3 ± 19.2	81.8 ± 19.5	77 ± 19.9
Age (y) at AD diagnosis	9.1 ± 16.1	5.1 ± 10.6	13 ± 20.1
Disease severity outcome			
EASI score	22 ± 15.5	23.7 ± 13.8	20.3 ± 17.8
SCORAD score	48.3 ± 19.4	49.4 ± 17.7	47.3 ± 22.1
IGA score	3.6 ± 0.8	3.8 ± 0.7	3.5 ± 0.9
POEM score	19.1 ± 7.6	18 ± 7.9	20.3 ± 7.7
DLQI score	13.1 ± 8.3	12.3 ± 9.3	14 ± 7.6
Disease severity			
Mild AD	2	0	2
Moderate AD	6	4	2
Severe AD	8	4	4
Blood eosinophils (10^6^/mL)	0.59 ± 0.53	0.65 ± 0.66	0.53 ± 0.39
Allergen extract for IDAC			
HDM	15	8	7
Alternaria	1	0	1

Data are presented as “n” or mean ± SD.

### Skin assessments before treatment

**Fig 2:**
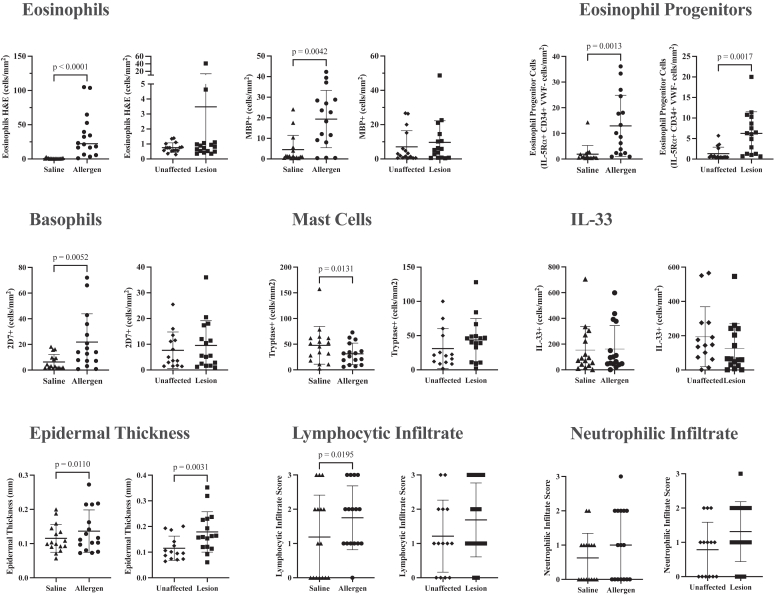
The effect of IDAC compared with saline control challenge on the accumulation of cells in skin biopsies sampled pretreatment at day 1, 24 hours after day 0 challenges, and inflammatory cells present in chronic lesions compared with unaffected skin collected pretreatment at day 1. All cells were measured in the papillary dermis, with the exception of IL-33^+^ cells, which were measured in the epidermis and papillary dermis of the skin. Epidermal thickness was measured in millimeters. Data are shown as raw values and mean ± SEM.

IDAC at day 0 pretreatment induced early and late cutaneous responses with wheal sizes of 1.93 ± 0.18 and 1.39 ± 0.19 mm^2^, respectively, and larger than control saline challenge wheal sizes of 0.48 ± 0.11 (*P* < .0001) and 0 ± 0 (*P* < .0001), respectively. In the papillary dermis at 24 hours post-IDAC, there was a significantly higher number of eosinophils measured by H&E (*P* < .0001) and by MBP immunoreactivity (*P* = .0042), as well as higher EoP cells (*P* = .0013) and basophils (*P* = .0052) compared with the saline-challenged control (Fig 2), in keeping with previously studied inflammatory reactions 24 hours post-IDAC.^23^ IDAC also increased epidermal thickness (*P* = .011) and the lymphocytic infiltrate score (*P* = .0195). The number of mast cells was higher in saline-challenged skin than in allergen-challenged skin (*P* = .0131). Compared with the papillary dermis of unaffected skin, lesional skin had a higher number of EoP cells (*P* = .0017) and a thicker epidermis (*P* = .0031) but no difference in the numbers of eosinophils, basophils, mast cells, lymphocytic or neutrophilic infiltrates, and IL-33^+^ cells (Fig 2). The levels of EoPs in skin lesions were significantly correlated with EASI (*r* = 0.53; *P* < .05) and SCORAD (*r* = 0.50; *P* < .05) scores (data not shown).

### The effect of prednisolone treatment on clinical outcomes

**Fig 3:**
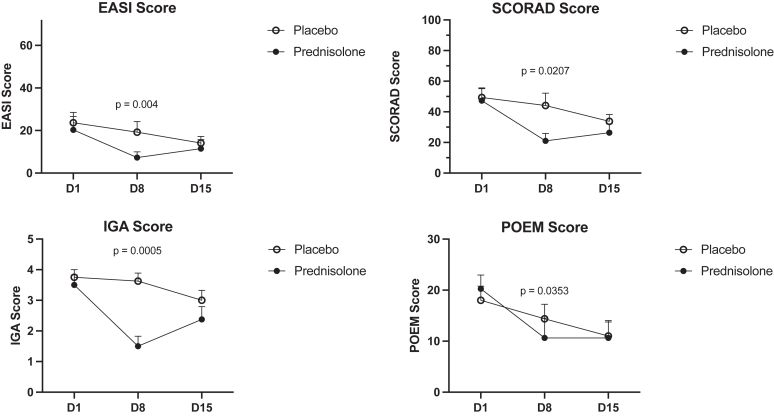
The effect of prednisolone compared with placebo on clinical outcomes including EASI, SCORAD, IGA, and POEM. Data are shown as the group mean ± SEM.

Prednisolone treatment of 0.75 mg/kg rapidly and significantly improved clinical disease severity assessed by EASI, SCORAD, IGA, and POEM (*P* < .05) by day 8 (Fig 3). These clinical effects were lost during subsequent tapering of prednisolone 0.5 to 0.25 mg/kg over the following week, and differences were no longer observed at day 15.

### The effect of prednisolone treatment on response to IDAC

**Fig 4:**
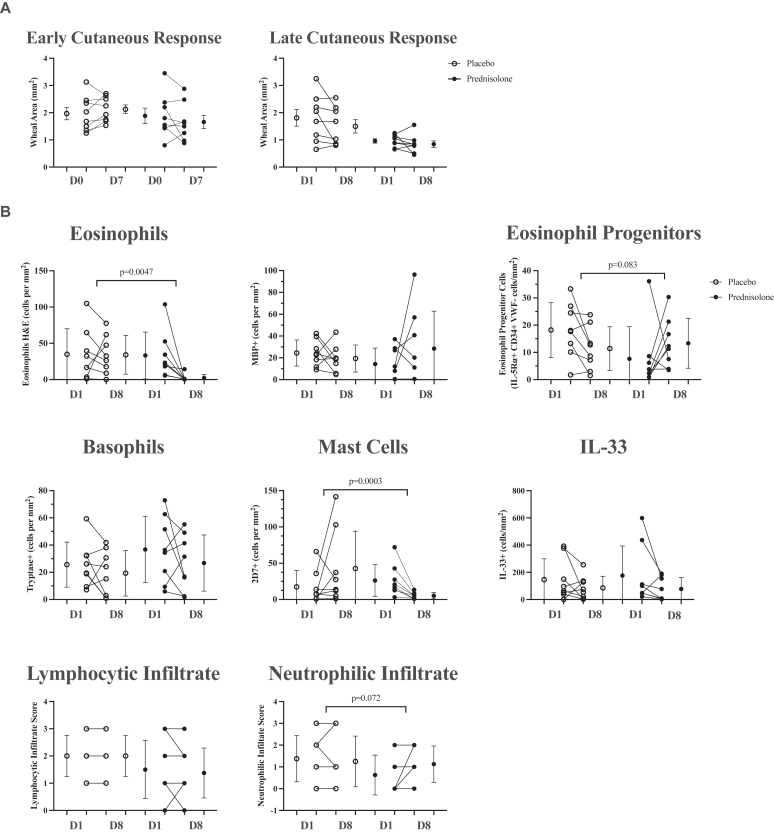
The effect of prednisolone compared with placebo on skin responses to IDAC. **A,** Skin wheal size (mm^2^) measured during the early cutaneous response (10 minutes postchallenge) at day 0 pretreatment and day 7 posttreatment and during the late cutaneous response (24 hours postchallenge) at day 1 pretreatment and day 8 posttreatment. **B,** Inflammatory cells measured in allergen-challenged skin collected 24 hours after challenge pretreatment at day 1 and posttreatment at day 8. All cells were measured in the papillary dermis except IL-33^+^, which was quantified in the papillary dermis plus epidermis. Data are shown as raw values and mean ± SEM.

Prednisolone did not alter the size of the IDAC-induced early or late cutaneous responses (Fig 4, *A*). However, prednisolone treatment significantly inhibited cellular inflammation measured in the papillary dermis of IDAC skin collected 24 hours after challenge, including the number of eosinophils (*P* = .0047) and basophils (*P* = .0003) (Fig 4, *B*). Cytokine measurements from biopsies collected 24 hours after IDAC demonstrated a reduction in IL-1β, IL-17A, and TGF-α levels in prednisolone compared with the placebo group (*P* < .05) (see Table E1 in this article’s Online Repository at www.jaci-global.org).

### The effect of prednisolone treatment on chronic AD skin lesions

There was no effect of prednisolone treatment on our measures of cellular inflammation nor any effect on epidermal thickness in the lesional skin (see Fig E1 in this article’s Online Repository at www.jaci-global.org). However, prednisolone significantly reduced the levels of IL-5, IL-9, IL-10, IL-13, TNF-α, TARC, and MIP-1β. There was a significant increase in SCF and IL-1α levels in lesional skin after prednisolone treatment (Table E1).

### Relationship between clinical scores and measures of inflammation

**Fig 5:**
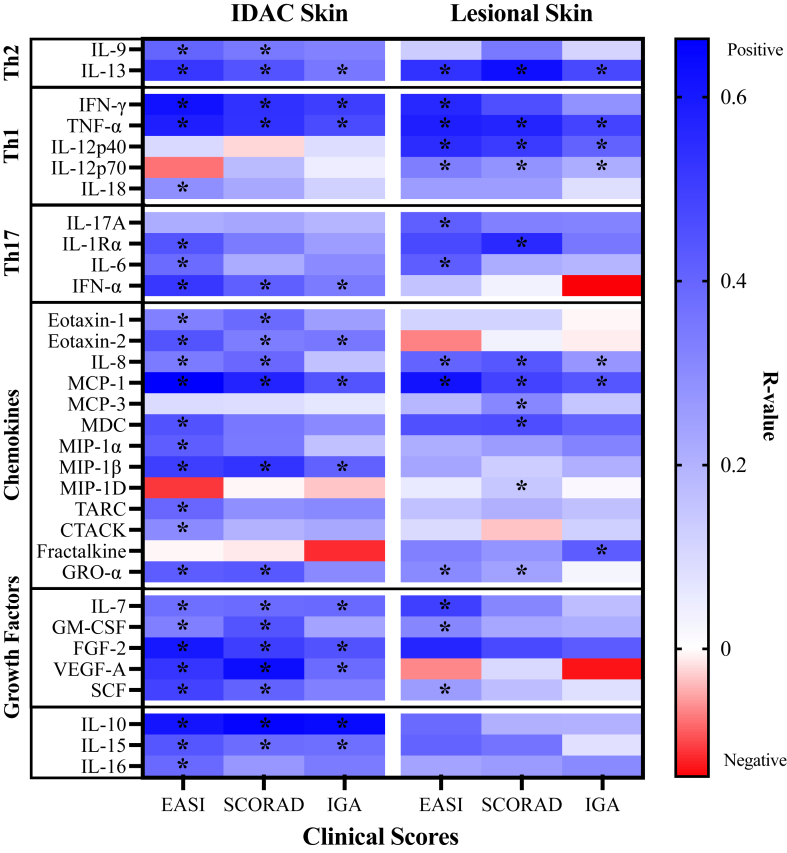
Heat map of the Pearson correlation from the multiple linear regression used to examine the associations between clinical disease scores (EASI, SCORAD, and IGA) and cytokines measured from the allergen challenge biopsy and the lesion biopsy from all study patients, sampled 24 hours after IDAC on day 1 and day 8 combined. ∗*P* values are overlaid on the corresponding cytokine.

To evaluate the association between improvements in clinical scores and changes in inflammatory measures, we performed multiple linear regression for each clinical score, using the raw values of all outcomes and time points. Several cytokines measured in lesional and IDAC skin were significantly associated with the clinician-determined scores (EASI, SCORAD, and IGA) (Fig 5) but not with the patient-determined scores (POEM or DLQI) (data not shown). Notably, the key cytokines IL-13, TNF-α, and MCP-1 demonstrated the same patterns of association in the lesional and IDAC skin, having strong positive correlations (*r* > 0.6) with EASI and SCORAD (Fig 5; see also Fig E2 in this article’s Online Repository at www.jaci-global.org). In the IDAC skin, we also observed a strong relationship with IL-10; moderate to strong positive associations (*r* values between 0.4 and 0.6) with IFN-γ, FGF-2, and VEGF-A (all *P* < .05); and weak to moderate associations (*r* values between 0.2 and 0.4) with IL-8, IL-9, IL-13, IL-15, IFN-α, eotaxin 1 and 2, MIP-1β, and SCF. Many of these associations were remarkably absent in the lesional biopsies (Fig E2). In the skin lesions, we observed distinct and significant positive associations between EASI, SCORAD, and IGA scores and levels of IL-12p40, IL-12p70, and MDC that were not present in the IDAC skin. Measures of eosinophils by H&E staining were positively and significantly associated with EASI, SCORAD, and IGA in the IDAC skin and with EASI in the lesional skin. In contrast, measures of mast cells and IL-33^+^ cells were positively and significantly associated with the patient-determined POEM and/or DLQI scores but not with the clinician-determined EASI, SCORAD, and IGA scores (see Fig E3 in this article’s Online Repository at www.jaci-global.org).

## Discussion

Acute treatment with oral corticosteroids is prescribed for patients who require rapid relief from severe AD flares.^30^ Oral corticosteroids should not be used for long-term treatment, and hence new therapeutic targets that exhibit similar effectiveness are needed. In this study, we examined histologic features and cytokine profiles in skin biopsy samples taken from an IDAC site at 24 hours postchallenge and a chronic lesion site. By evaluating both early-induced and chronic-persistent inflammation in these biopsies, we aimed to determine whether immunologic patterns were similar and consistent with clinical outcomes following prednisolone treatment. We hypothesized that there would be differences between allergen-challenged and lesional skin, given the histologic differences and immune shifts that occur from acute to chronic lesions.^31^ In this study, we demonstrated that prednisolone treatment significantly improved AD severity, as measured by clinical scores (EASI, SCORAD, and IGA) and the patient-oriented score (POEM), and that this improvement was accompanied by changes in T_H_2 and T_H_17 inflammation in lesional and allergen-challenged skin and in T_H_1 inflammation in lesional skin. To our knowledge, this is the first study to investigate the cellular changes in both allergen-challenged and lesional skin after acute corticosteroid treatment and the first to describe the association of steroid-induced shifts in clinical outcomes with measures of inflammation in the skin of patients with AD.

We first characterized skin lesions of patients with AD by comparing them to unaffected skin. In the papillary dermis, we found significantly higher levels of EoPs, which interestingly showed a moderate positive relationship with EASI scores. EoPs have been reported to have potent effector functions, including the release of T_H_2 cytokines, such as IL-13.^32^ Furthermore, EoPs express IL-4Rα and are therefore targets of dupilumab, which is effective for the treatment of moderate to severe AD.^9^ EoP levels were also significantly higher in allergen-challenged skin compared with saline control, along with elevated numbers of eosinophils measured by H&E and by MBP immunoreactivity, and basophils (all *P* < .05).

We then compared the effects of prednisolone treatment on immune pathways in allergen-challenged and lesional skin. Findings from our study confirm that cytokines across several T-cell subsets are elevated in patients with AD. In skin lesions, prednisolone treatment significantly reduced levels of most T_H_2 cytokines (IL-5, IL-9, and IL-13, but not IL-4), many chemokines, and the T_H_1 cytokine TNF-α, without altering the number of inflammatory cells. In contrast, in the IDAC skin, prednisolone treatment significantly reduced levels of the migratory eosinophils and basophils, but had limited effects on cytokine levels. Furthermore, the cytokines inhibited by prednisolone in skin lesions had virtually no overlap with cytokines inhibited in allergen-challenged skin. This selective effect could be explained by differential inflammatory pathways driving acute versus chronic AD skin, with the latter associated with structural changes, as evidenced by a thicker epidermis, and perhaps requiring prolonged treatment to resolve these transformations. Previous comparisons of acute and chronic AD skin using RNA-sequencing analysis reported 74% overlap in dysregulated genes, indicating that chronic inflammation represents an amplification rather than a fundamental shift in a distinct immune profile.^31^ The acute response in skin is dominated by keratinocyte alarmin release, ILC2 activation, T_H_2 polarization, mast cell/eosinophil recruitment, and amplification of T_H_2 cytokines (notably IL-4, IL-5, IL-13, and IL-33). Clinically, although our small study showed a significant effect of prednisolone treatment on measures of disease severity, it did not show an effect of prednisolone on the development of skin wheals measured 10 minutes and 24 hours after intradermal challenge, reflecting the lack of impact on acute mediator release from mast cells after allergen challenge.

Sustained T_H_2 inflammation has been shown to impair barrier function and dendritic cell maturation, thereby promoting the release of IL-12 and IL-23 and driving T_H_1 (IFN-γ)/T_H_17 (IL-17A) differentiation.^33^ Chronic maintenance of skin lesions is marked by mixed T_H_2 (sustained itch and IgE levels), T_H_1 (mediating delayed-type hypersensitivity), T_H_17/IL-36 (contributing to neutrophilic inflammation), and T_H_22/IL-22 infiltration (promoting keratinocyte hyperplasia, lichenification, and impaired differentiation).^31^ We observed a treatment effect across T_H_1, T_H_2, and T_H_17 cytokines, providing evidence that prednisolone inhibits a broad range of inflammatory pathways. Most of the anti-inflammatory and immunosuppressive actions of glucocorticoids are attributable to the direct or indirect transcriptional effects of glucocorticoids, which alter the transcription of numerous genes in leukocytes. Early studies using blood cell cultures demonstrated that dexamethasone inhibited the production of both T_H_1 and T_H_2 cytokines after LPS stimulation,^34^ as well as IL-12 production, thereby also limiting development of T_H_1 cells.^35^ Our clinical trial showed no effect of glucocorticoids on IL-12, whereas IL-10, which can suppress both T_H_1- and T_H_2-driven inflammation, was significantly reduced in lesions. Previous studies have found that in patients with AD, there is impaired IL-10 production, and that IL-10 is negatively associated with clinical disease severity measures.^36^^,^^37^ Hence, the overall impact of steroid treatment is likely context-dependent and effective across a broad range of patients, whereas cytokine-specific biologics are effective only in phenotypic subsets.

Despite seemingly different regulation of inflammation by prednisolone treatment in AD skin lesions versus allergen-challenged skin, we found remarkable similarities in the association of clinical outcomes with levels of specific cytokines measured in both lesional and IDAC skin. IL-13, IFN-γ, TNF-α, IL-8, and MCP-1 levels in lesions and in IDAC skin were positively associated with clinical outcomes (EASI, SCORAD, and IGA). Together, our findings suggest that the regulation of these specific cytokines contributes to the skin characteristics used for clinical assessments of AD severity. We also observed some consistent associations between cell numbers and clinical outcomes. In allergen-challenged skin, EoPs and basophils were negatively associated with all clinical outcomes. In contrast, mast cells in allergen-challenged and lesional skin were positively and significantly associated with patient-reported outcomes, POEM, and DLQI. Mast cells are located near peripheral nerves and release mediators of itch, including histamine, thereby directly modulating neuroimmune interactions and enhancing itch,^6^ which correlates with patients’ scores on POEM and DLQI. The association of mast cells with more subjective patient scores but not with the objective EASI and SCORAD clinical measurements highlights the role of mast cell mediators in driving AD symptoms.

Interestingly, total eosinophils measured by H&E were positively associated with clinical outcomes, whereas MBP^+^ eosinophils were not. Our understanding of eosinophil phenotypes is evolving,^14^^,^^38^ and subsets of eosinophils with different functions have not been taken into consideration in this study, nor in the interpretation of clinical trials demonstrating the failure of IL-5/IL-5Rα biologics in patients with AD.^13^^,^^14^ Clinical trials targeting TSLP,^17^ IL-33,^39^ and IgE^15^ have also failed in AD, suggesting that, unlike asthma, these inflammatory pathways are not key drivers of this disease. We saw no effect of prednisolone on IL-33^+^ cells or IL-33 cytokine levels in lesions or IDAC skin. These data support findings from a clinical trial showing that anti–IL-33 treatment did not improve EASI scores, blood eosinophils, or serum IL-5 and CCL13 levels in patients with AD.^39^ Our data contribute to the growing evidence that the role of alarmin cytokines, including IL-33, lies in initiating rather than maintaining disease.

The positive associations we observed between clinical outcomes and IL-13 are validated by the demonstrated efficacy of dupilumab and lebrikizumab for treatment of AD,^40^^,^^41^ and the positive associations observed between clinical outcomes and cytokines that are regulated by JAK/STAT pathways such as IL-7, GM-CSF, IFN-α, and IFN-γ also display pathways through which JAK inhibition improves disease severity in AD.^42^, ^43^, ^44^, ^45^ Our data point to other cytokine pathways that drive AD severity, such as TNF-α, which, when blocked, can improve or worsen AD,^46^^,^^47^ as well as to pathways that have not been tested, such as MCP-1 and FGF-2.

There are limitations to interpreting our data. We used a model of IDAC to examine immunologic pathways activated by allergen exposure in a controlled setting. Although this experimental model allows us to investigate reproducible T_H_2-dominant pathways, it may skew cytokines toward a T_H_2/T_H_22/T_H_17 axis. The clinical relevance for patients with AD may be limited.^48^^,^^49^ An example of this variable effect is observed in our IDAC data on IL-10: a significant increase in IL-10 in the IDAC skin was associated with disease severity. Interestingly, although IL-10 production in response to allergens is generally attenuated in patients with AD, a study in children found that house dust mite specifically induced increased IL-10 responses.^50^ This example illustrates some of the challenges with our experimental model. Although lymphocytic and neutrophilic infiltrates were significantly associated with multiple clinical outcomes in our patients, this association may be partly attributable to the narrow variability of the 4-point scoring system.^24^ Underestimation in our measures of eosinophils, basophils, and mast cells detected by MBP, basogranulin, and tryptase, respectively, could occur because of cell degranulation, which occurs postactivation.^51^, ^52^, ^53^ For example, significantly more mast cells identified by tryptase immunopositivity were present in the saline control biopsy compared with the allergen challenge biopsy, and this also could reflect the rapid release of preformed mediators and postallergen degranulation state of mast cells, leading to underestimation of these cells using intracellular tryptase as a marker.^54^

The data from our study advance the understanding of AD pathobiology by demonstrating how oral prednisolone exerts differential immunomodulatory effects in lesional versus allergen-challenged skin, resulting in rapid clinical improvement in measures of disease severity. While confirming steroid suppression of T_H_2 cytokines such as IL-5, IL-9, and IL-13 in chronic lesions, we also observed reductions in proinflammatory mediators including TNF-α, IFN-γ, IL-8, and MCP-1, highlighting that glucocorticoids affect a broader network of immune pathways in AD skin than previously appreciated. The identification of these distinct associations between cytokines and clinical outcomes can be used to support the development of additional biologic targets for therapeutic intervention in patients with AD.

**Key messages**

• **In allergen-challenged skin, we observed an effect of prednisolone on proinflammatory migrating cells, whereas in chronic skin lesions we observed an effect of prednisolone across a broad range of inflammatory pathways including T_H_1, T_H_2, and T_H_17 cytokines.**
• **In both lesional and IDAC skin, the AD clinical severity outcomes (EASI, SCORAD, and IGA) demonstrated significant positive associations with levels of specific cytokines including IL-13, IFN-γ, TNF-α, IL-8, and MCP-1.**

## Disclosure statement

Disclosure of potential conflict of interest: The authors declare that they have no relevant conflicts of interest.
