# Supplementary material for: Pathobiology of atopic dermatitis and association with disease severity after acute oral steroid treatment
Source: J Allergy Clin Immunol Glob. 2026 Mar 28;5(4):100696. doi: 10.1016/j.jacig.2026.100696 (PMC13123604; doi:10.1016/j.jacig.2026.100696)
Supplement: Supplementary Figs and Tables [file mmc1.docx]

**ONLINE SUPPLEMENT**

**PATIENT ELIGIBILITY**

16 patients between 18-65 years of age with a diagnosis of moderate to severe atopic dermatitis as determined by EASI, SCORAD and IGA scores were recruited. Patients underwent a screening visit 29-14 days prior to initiation of the study to screen for inclusion and exclusion criteria (Table 1). Inclusion criteria included a positive skin-prick test to common aeroallergens; a positive allergen-specific IgE blood RAST test, and the development of a positive late cutaneous response to intradermal allergen challenge. Patients were excluded if treatment with an investigational drug occurred 8 weeks or 5 half-lives before baseline visit (D0); treatment with immunosuppressive or immunomodulating drugs 4 weeks prior to baseline visit (D0); any prior treatment with biologics; the initiation of prescription moisturizers or moisturizers containing additives such as ceramide, hyaluronic acid, urea, or filaggrin degradation products during the screening period (day-28 to day 0) (patients were allowed to continue use of stable doses of such moisturizers if initiated before the screening visit (day-28)); regular use of tanning booths 4 weeks before baseline visit (day 0); active chronic or acute infection requiring treatment with systemic antibiotics, antivirals, antiparasitics, antiprotozoals, or antifungals 2 weeks before the baseline visit (day 0), or superficial skin infections 1 week before the baseline visit (day 0). Patients were excluded if known or suspected history of immunosuppression; including history of invasive opportunistic infections; history of human immunodeficiency virus (HIV) infection or history of hepatitis B or hepatitis C infection; the presence of any skin comorbidities or other concomitant illness(es) including psychological conditions that, in the investigator’s judgment, would adversely affect the patient’s participation in the study.

**STUDY PROCEDURES**

**Run-in Period**

Prior to the start of the study, patients discontinued use of systemic immunosuppressant’s four weeks before day 0. Sixteen days before day 0, patients were given a low dose of oral prednisolone (0.25 mg/kg) for 8 days and discontinued the use of prednisolone thereafter. Five days before day 0, patients stopped the use of any antihistamine or doxepin.

**Allergen Prick Test and Intradermal Challenges (Day 0 and Day 7)**

Skin prick tests and intradermal allergen/saline challenges were conducted pre-treatment at D0 and post-treatment at D7. Standard allergen extracts used in the skin prick test included ragweed, trees, grass, dog, cat, horse, dust mites (*Dermatophagoides* farinae and *Dermatophagoides pteronyssinus*), alternaria, and aspergillus. Allergen extracts manufactured following Good Manufacturing Practice guidelines, were selected, prepared by staff and administered to the subjects. A positive control (1 mg/mL histamine) and a negative control (diluent) were also applied to the skin. After 10 minutes, the size of the wheal was measured. A skin wheal greater than 2 × 2 mm was regarded as a positive reaction, provided that the positive and negative controls were appropriately positive (histamine) and negative (diluent), respectively. Based on skin prick and allergen specific IgE blood RAST test, the investigator chose an allergen for skin prick titration. The selected allergen was diluted 2, 4, 8, 16, 32, 64, 128, and 256-fold with normal saline and an additional skin prick test was performed. After 10 minutes, the size of the wheal was measured. The dilution that resulted in a wheal size of 3x3 mm was selected for intradermal allergen challenge. Two intradermal allergen challenges were performed using a volume of 100 μL intradermally in one standardized location on the patient’s back. A saline control with a volume of 100 μL was injected intradermally in one standardized location on the patient’s back. The acute response was measured 10 minutes post intradermal allergen challenge. 24 hours post challenge, the size of the late cutaneous response was measured.

**Skin Biopsy (Day 1 and Day 8)**

Punch biopsies were obtained from the site of the intradermal allergen and saline challenges using a sterile 4 mm skin punch by applying and twisting until the blade of the skin punch has pierced the skin. The biopsy was removed using sterile forceps and a scalpel. These samples were processed for histologic examination of inflammatory cells, including eosinophils, mast cells, basophils, and supernatant cytokine levels. Before a biopsy was taken, the skin was thoroughly cleaned, and local anesthetic (2% lidocaine) is injected to numb the skin. The site of skin biopsies and excision was sutured and covered with a sterile bandage. Patients were instructed how to keep the site clean, and sutures were removed at the next study visit.

**Randomization (Day 1)**

Patients were randomized 1:1 to receive either prednisolone or placebo for 15 days. Prednisolone dosage was tapered using the following regium: 5 days at 0.75 mg/kg, 5 days at 0.5 mg/kg and 5 days at 0.25 mg/kg.

**Clinical Measurements (Day 0, Day 7, Day 14)**

The investigator assessed clinical measurements included Eczema Area and Severity Index (EASI), Severity Scoring of Atopic Dermatitis Index (SCORAD), and the Investigators Global Assessment (IGA). The patients reported assessments included the questionnaires Dermatology Life Quality Index (DLQI) and Patient Oriented Eczema Measure (POEM).

**LABORATORY PROCEDURES**

**Immunofluorescence and immunohistochemistry (IF and IHC) and Imaging**

Tissues were formalin-fixed, embedded into paraffin blocks and H&E stained by the McMaster Core Histology Laboratory Facility. Tissue sections were stained by indirect immunofluorescence using antibodies to eosinophil cationic protein (EG2), major basic protein (MBP), CD125, eosinophil progenitor cells (CD34+ CD125+ Von Willebrand-), basophils (2D7) and mast cells (tryptase). Paraffin-embedded tissues were cut into 5um sections and mounted on frosted positively charged glass slides. Sections were deparaffinized in xylene and rehydrated with subsequent reductions in decreasing concentration of ethanol and subjected to heat-induced antigen retrieval in sodium citrate buffer (pH=6) under pressure for 5 minutes. Permeabilization of cells was performed using 0.2% trition X-100 for 30 minutes. For eosinophil progenitor cells, cells were identified as being CD34+ CD125+ and Von Willebrand negative. Staining was performed using 3% normal horse blocking serum in PBS for 30 minutes and incubated with primary antibody goat anti-human CD125 (PA5-47340; Invitrogen, MS, United States; dilution 1:250) overnight at 4°C and detected using a donkey anti-goat Alexa Fluor 488 (ab6881; abcam, Cambridge, United Kingdom) for 1 hour at room temperature. Followed by 1.5% normal horse blocking serum and 1.5% normal goat blocking serum in PBS for 30 minutes and incubated with primary antibody mouse anti-human CD34 (ab8536; abcam, Cambridge, United Kingdom; dilution 1:100) overnight at 4°C and detected using a goat anti-rabbit Alexa Fluor 594 (R37121; Invitrogen, MS, United States) for 1 hour at room temperature. And finally, using 1.5% normal horse blocking serum and 1.5% normal goat blocking serum in PBS for 30 minutes and incubated with primary antibody rabbit anti-human Von Willebrand (ab6994; abcam, Cambridge, United Kingdom; dilution 1:200) overnight at 4°C and detected using a goat anti-rabbit Alexa Fluor 647 (A21244; Invitrogen, MS, United States) for 1 hour at room temperature. For assessing mature eosinophils, MBP staining was performed by using 1.5% normal goat blocking serum and 1.5% normal horse blocking serum in PBS for 30 minutes and incubated with primary antibody rabbit anti-human MBP (ab187523; abcam, Cambridge, United Kingdom; dilution1:200) overnight at 4°C and detected using a goat anti-mouse Alexa Fluor 647 (A21244; Invitrogen, MS, United States) for 1 hour at room temperature. For the basophil, mast cell and IL-33 stains, tissues were stained with antibodies to IL-33, 2D7 (basophils), and tryptase (mast cells). IL-33 staining was performed by using 3% normal donkey blocking serum in PBS for 30 minutes and incubated with primary antibody goat anti-human IL-33 (PA5-47006; Invitrogen, MS, United Sates; dilution 1:40) overnight at 4°C and detected using a donkey anti-goat Alexa Fluor 594 (A11058; Invitrogen, MS, United States) for 1 hour at room temperature. Followed by 1.5% normal horse blocking serum and 1.5% normal goat blocking serum in PBS for 30 minutes and incubated with primary antibody mouse anti-human 2D7 (ab155577; abcam, Cambridge, United Kingdom; dilution 1:250) overnight at 4°C and detected using a goat anti-mouse Alexa Fluor 488 (A11001; Invitrogen, MS, United States) for 1 hour at room temperature. And finally, using 1.5% normal horse blocking serum and 1.5% normal goat blocking serum in PBS for 30 minutes and incubated with primary antibody rabbit anti-human tryptase (ab134931; abcam, Cambridge, United Kingdom; dilution 1:250) overnight at 4°C and detected using a goat anti-rabbit Alexa Fluor 647 (A21244; Invitrogen, MS, United States) for 1 hour at room temperature. Tissues were counter stained with DAPI and cover slipped. Tissues were imaged using the Light Microscope Basics Nikon Upright Eclipse Ni-U and analyzed using the NIS-Elements program. Cells of interest expressing any of the studied markers were counted manually and divided by the area of the region of interest and expressed as number per mm^2^). The precent changes from pre to post treatment were compared between the benralizumab and placebo groups using Mann Whitney U-test.

**Skin Biopsy Supernatant Preparation and Mediator Measurements**

The weight of the allergen challenge biopsy was obtained. dPBS was added to the biopsy according to the following formula: (weight of biopsy (g) x 1.5 mL)/ 0.1g. The biopsy was minced into a goopy paste using a scalpel and dissection scissors. Once sufficiently minced, the biopsy paste was placed in a 37°C shaking incubator for 120 minutes. Supernatant was transferred into a fresh tube and centrifuged to remove excess tissue while the remaining solid tissue was disposed of. The supernatants were removed and divided into 1.5 mL Eppendorf’s and centrifuged again. 247.5 uL of superannuants were aliquoted into new Eppendorf containing 2.5 uL of Sigma Protease Inhibitor and stored at -80°C. Cytokines and chemokines were measured using Eve Technologies 65-cytokine ELISA.

**Supplementary Figure** **1**. The effect of prednisolone compared to placebo on lesional skin in the papillary dermis of skin from all study patients, sampled 24 hours after challenge on day 1 and day 8. Eosinophils were defined by H&E staining, and MBP+ cells by immunofluorescence staining. Eosinophil progenitors (EoP) were defined as CD45+CD34+CD125+ cells by immunofluorescence staining. Basophils were defined as 2D7+ cells by immunofluorescence staining. Mast cells were defined as tryptase+ by immunofluorescence staining. IL-33+ cells were measured by immunofluorescence in both the epidermis and papillary dermis of the skin. Lymphocytic and neutrophilic infiltration scores were measured on a scale of 0 (absent) to 3 (severe). Individual and mean (SEM) data points are shown. The delta change from pre- to post-treatment measurements was compared between the prednisolone and placebo groups and analyzed by the Mann-Whitney U test.

**Supplementary Figure 2**. A heat map of the Pearson correlation from the multiple linear regression used to examine the associations between clinical disease scores (EASI, SCORAD, and IGA) and cytokines measured from (A) the allergen challenge biopsy and (B) the lesion biopsy from all study patients, sampled 24 hours after IDAC on day 1 and day 8 combined.

**Supplementary Figure 3**. A heat map of the Pearson correlation from the multiple linear regression used to examine the associations between clinical disease scores (EASI, SCORAD, IGA, POEM, and DLQI) and cell counts measured from (A) the allergen challenge biopsy and (B) the lesion biopsy from all study patients, sampled 24 hours after challenge on day 1 and day 8 combined. * P-values are overlaid on the corresponding cytokine. IDAC: Intradermal allergen challenge.

**Supplementary Table 1**. The effect of prednisolone compared to placebo on cytokine levels from intradermal allergen-challenged and lesion biopsy supernatants at day 1 (pre-treatment) and day 8 (post-treatment). Data is reported as mean ±SEM. Eleven of the 65 cytokines measured (6CKine, Eotaxin-3, G-CSF, IL-21, IL-28, LIF, MCP-2, MCP-4, SDF-1α + β, TPO, TSLP) were below the lower limit of detection and data are not included. The percent change was compared using the Mann-Whitney U-test, and p-values are uncorrected. *p≤0.05; ↓= reduced after prednisolone treatment; ↑= increased after prednisolone treatment.

| Cytokine  (pg/mL) | Allergen Challenge | | | | | Lesion | | | | |
| --- | --- | --- | --- | --- | --- | --- | --- | --- | --- | --- |
|  | Prednisolone  day 1 day 8 | | Placebo  day 1 day 8 | | P-value | Prednisolone  day 1 day 8 | | Placebo  day 1 day 8 | | P-value |
| Th2 | | | | | | | | | | |
| IL-4 | 5.78±1.6 | 5.15±1.8 | 13.76±6.9 | 15.95±5.2 | 0.336 | 5.07±0.7 | 4.82±0.9 | 5.3±1.1 | 4.9±0.9 | 0.636 |
| IL-5 | 0.55±0.4 | 0.36 ±0.2 | 3.53 ±1.8 | 4.81 ±2.2 | 0.344 | 0.49 ±0.1 | 0.17 ±0.08 | 0.27 ±0.09 | 0.28 ±0.08 | **0.001* ↓** |
| IL-9 | 0.53±0.1 | 0.46 ±0.2 | 4.52 ±2.5 | 6.28 ±3.5 | **0.05* ↓** | 1.43 ±0.5 | 0.23 ±0.03 | 0.42 ±0.1 | 0.44 ±0.2 | **<0.001* ↓** |
| IL-13 | 1.23±0.5 | 0.89 ±0.4 | 2.6 ±1.1 | 3.64 ±1.8 | 0.105 | 3.04 ±0.7 | 0.61 ±0.2 | 1.86 ±0.7 | 1.78 ±0.6 | **<0.001*↓** |
| Th1 | | | | | | | | | | |
| IFN-γ | 1.43±0.2 | 1.38 ±0.3 | 1.32 ±0.2 | 1.61 ±0.3 | 0.279 | 2.16 ±0.3 | 1.44 ±0.3 | 1.56 ±0.2 | 1.53 ±0.2 | 0.074 |
| TNF-α | 1.34±0.6 | 0.88 ±0.6 | 2.31 ±0.9 | 1.63 ±0.6 | 0.505 | 3.28 ±0.9 | 1.24 ±0.2 | 1.85 ±0.6 | 1.93 ±0.5 | **0.01* ↓** |
| IL-2 | 0.34±0.1 | 0.3 ±0.09 | 0.46 ±0.1 | 0.51 ±0.09 | 0.798 | 0.56 ±0.09 | 0.52 ±0.05 | 0.41 ±0.06 | 0.52 ±0.06 | 0.382 |
| IL-12p40 | 2.38 ±0.3 | 2.97 ±0.4 | 1.98 ±0.6 | 2.47 ±0.4 | 0.613 | 6.82 ±1.8 | 4.98 ±0.9 | 4.41 ±0.8 | 4.96 ±0.6 | 0.382 |
| IL-12p70 | 0.6 ±0.1 | 0.52 ±0.8 | 0.66 ±0.1 | 0.73 ±0.08 | 0.662 | 0.72 ±0.09 | 0.52 ±0.06 | 0.91 ±0.07 | 0.81 ±0.1 | 0.674 |
| IL-18 | 194.22 ±24 | 150.39 ±31.7 | 127.08 ±32 | 170 ±39 | 0.065 | 909.8 ±126.8 | 853.4 ±201.2 | 795.5 ±144.8 | 737.4 ±98.6 | 0.721 |
| sCD40L | 0.44 ±0.05 | 0.71 ±0.1 | 0.44 ±0.1 | 0.58 ±0.07 | 0.751 | 0.76 ±0.1 | 0.66 ±0.1 | 0.52 ±0.1 | 0.6 ±0.07 | 0.207 |
| Th17 | | | | | | | | | | |
| il-17a | 0.8 ±0.07 | 0.64 ±0.08 | 0.89 ±0.1 | 1.21 ±0.2 | **0.038*↓** | 1.03 ±0.1 | 0.97 ±0.1 | 0.98 ±0.08 | 0.91 ±0.08 | 1 |
| IL-23 | 14.44 ±3.7 | 23.85 ±8 | 27 ±8.9 | 17.32 ±4.3 | 0.417 | 13.89 ±1.9 | 19.13 ±4 | 12.63 ±2.7 | 15.67 ±3 | 0.209 |
| IL-33 | 12.09 ±1.7 | 14.31 ±4.2 | 8.9 ±2.6 | 9.2 ±2.5 | 0.431 | 122.13 ±26.3 | 132.04 ±22.2 | 135.86 ±22.5 | 141.96 ±26.7 | 0.382 |
| IL-1Rα | 1356.9 ±234.6 | 1148.6 ±297 | 883 ±235.6 | 1212.8 ±352 | 0.195 | 8425 ±1090 | 7500 ±1274.6 | 6821.8 ±980.2 | 7461.5 ±980 | 0.279 |
| IL-1β | 0.61 ±0.1 | 0.38 ±0.04 | 0.61 ±0.2 | 0.95 ±0.4 | **<0.001*↓** | 1.18 ±0.2 | 1.89 ±0.7 | 1.11 ±0.2 | 0.95 ±0.2 | 0.318 |
| IL-6 | 7.82 ±2.8 | 12.96 ±8.8 | 9.99 ±3.4 | 4.33 ±1.2 | 0.442 | 142.43 ±57.3 | 106.52 ±32.6 | 56.8 ±9 | 76.41 ±29 | 0.878 |
| IFN-α | 2.58±0.5 | 2.77±0.5 | 2.93±0.3 | 3.82 ±0.9 | 0.867 | 4.99 ±0.5 | 4.14 ±0.7 | 4.93 ±0.4 | 4.52 ±0.6 | 0.563 |
| TNF-β | 0.49 ±0.08 | 0.51 ±0.06 | 0.57 ±0.08 | 0.46 ±0.1 | 0.492 | 0.93 ±0.1 | 0.75 ±0.1 | 0.42 ±0.1 | 0.48 ±0.07 | 0.074 |
| TRAIL | 0.67 ±0.05 | 0.59 ±0.06 | 0.68 ±0.03 | 0.6 ±0.03 | 0.959 | 1.2 ±0.4 | 2.33 ±1.6 | 0.98 ±0.2 | 1.6 ±0.6 | 0.645 |
| ChemokineS | | | | | | | | | | |
| Eotaxin-1 | 3.5 ±0.8 | 4.4 ±0.5 | 5.19 ±0.9 | 4.32 ±0.4 | 0.293 | 4.94 ±0.6 | 5.28 ±0.4 | 4.91 ±0.5 | 4.69 ±0.6 | 0.346 |
| Eotaxin-2 | 8.67 ±2.5 | 7.81 ±3.6 | 12.11 ±3.2 | 14.28 ±3.2 | 0.156 | 22.43 ±11 | 10.85 ±4.5 | 8.67 ±1.5 | 14 ±3.7 | **0.021*↓** |
| IL-8 | 38.8 ±19.2 | 26.25 ±9.6 | 38.5 ±12.3 | 32.6 ±11.7 | 0.574 | 20.92 ±6.7 | 16.46 ±6.9 | 11.17 ±3.9 | 13.98 ±5.8 | 0.798 |
| IP-10 | 79.72 ±42.3 | 135.24 ±100.7 | 56.77 ±30.5 | 50.05 ±26.8 | 0.234 | 48.3 ±41.4 | 6.89 ±2.7 | 6.02 ±1.5 | 26.89 ±21.7 | 0.065 |
| MCP-1 | 190 ±74 | 126.22 ±59.3 | 190.7 ±51.3 | 149.63 ±43.7 | 0.645 | 435.2 ±177.5 | 236.5 ±68.2 | 217.9 ±57.6 | 219.5 ±59 | 0.645 |
| MCP-3 | 7.34 ±1 | 7.59 ±1 | 6.83 ±0.6 | 6.94 ±1 | 0.878 | 10.26 ±0.8 | 8.01 ±0.8 | 8.83 ±0.7 | 6.82 ±1.1 | 0.793 |
| MDC | 86.6 ±4.2 | 68 ±8.8 | 67.03 ±11.4 | 81.6 ±12.1 | 0.13 | 348.7 ±79.3 | 153.2 ±21 | 211.43 ±35.3 | 245 ±38.1 | 0.065 |
| MIP-1α | 2.22 ±0.4 | 1.92 ±0.33 | 2.48 ±0.32 | 2.63 ±0.63 | 0.753 | 2.62 ±0.3 | 1.62 ±0.4 | 2.33 ±0.2 | 2.01 ±0.3 | 0.156 |
| MIP-1β | 6.55 ±1.4 | 5.61 ±0.9 | 9.57 ±2.2 | 9.55 ±1.2 | 0.574 | 5.76 ±0.5 | 4.29 ±0.76 | 5.5 ±0.6 | 6.28 ±0.5 | **0.013*↓** |
| mip-1d | 4 ±0.2 | 3.57 ±0.5 | 3.75 ±0.6 | 3.23 ±0.3 | 0.875 | 3.08 ±0.4 | 2.77 ±0.3 | 3.44 ±0.3 | 2.97 ±0.3 | 0.713 |
| TARC | 6.66 ±3.4 | 5.51 ±3.4 | 44.3 ±18.4 | 43.8±17.9 | 0.13 | 3.12 ±1.1 | 0.48 ±0.12 | 0.86 ±0.23 | 1.82 ±1 | **0.007*↓** |
| CTACK | 13.53 ±2.6 | 12.65 ±2.1 | 29 ±8.3 | 27.7 ±8.4 | 0.959 | 30.54 ±7.6 | 16.81 ±3.4 | 28.57 ±8.6 | 81.43 ±51 | 0.442 |
| ENA-78 | 6.43 ±0.6 | 4.66 ±0.1 | 4.09 ±0.5 | 4.75 ±1 | 0.188 | 5.13 ±0.8 | 6.42 ±1.2 | 5.35 ±0.8 | 4.88 ±0.5 | 0.186 |
| Fractalkine | 11.68 ±2 | 13.13 ±2.1 | 10.43 ±2.1 | 14.16 ±2 | 1 | 19.8 ±1.7 | 13.03 ±2.2 | 15.59 ±2 | 17.14 ±1.7 | 0.207 |
| GRO-α | 57.45 ±17.6 | 53.26 ±14.6 | 51.6 ±10 | 60.77 ±18.4 | 0.721 | 80.34 ±14.6 | 69.7 ±14.5 | 51 ±11 | 58.36 ±7.1 | 0.248 |
| I-309 | 0.74 ±0.4 | 0.24 ±0.09 | 0.49 ±0.2 | 0.61 ±0.3 | 0.382 | 1.65 ±0.6 | 0.47 ±0.2 | 1.65 ±1 | 1.31 ±0.6 | **0.05*↓** |
| RANTES | 552 ±148 | 495.6 ±195 | 430.5 ±162 | 343.6 ±56 | 0.505 | 187.9 ±52 | 182.04 ±40 | 101.6 ±17.3 | 222 ±72 | 0.505 |
| BCA-1 | 0.16 ±0.03 | 0.11 ±0.03 | 0.15 ±0.02 | 0.11 ±0.03 | 0.959 | 0.31 ±0.05 | 0.22 ±0.04 | 0.15 ±0.04 | 0.27 ±0.07 | 0.059 |
| 6Ckine | 8.97 ±0.7 | 10.7 ±2.7 | 13.73 ±2.1 | 19.94 ±7.5 | 1 | 32.92 ±9.2 | 14.68 ±4.2 | 28.32 ±7.1 | 33.51 ±5.9 | 0.105 |
| Growth Factors | | | | | | | | | | |
| IL-3 | 0.48 ±0.06 | 0.47 ±0.1 | 0.32 ±0.07 | 0.43 ±0.07 | 0.574 | 0.4 ±0.06 | 0.36 ±0.1 | 0.43 ±0.05 | 0.31 ±0.05 | 1 |
| IL-7 | 0.68 ±0.06 | 0.62 ±0.06 | 0.75 ±0.1 | 0.75 ±0.1 | 0.195 | 1.39 ±0.3 | 1.16 ±0.1 | 1.34 ±0.1 | 1.13 ±0.1 | 0.878 |
| GM-CSF | 1.22 ±0.3 | 1.05 ±0.2 | 1.74 ±0.3 | 1.49 ±0.2 | 0.955 | 1.24 ±0.2 | 1.1 ±0.2 | 1.07 ±0.2 | 1.05 ±0.2 | 0.959 |
| EGf | 2.49 ±0.6 | 3.71 ±1.8 | 2.04 ±0.5 | 1.21 ±0.5 | 0.156 | 2.82 ±0.7 | 4.43 ±1 | 2.27 ±0.4 | 3.67 ±0.9 | 0.721 |
| FGF-2 | 246.5 ±57 | 280.7 ±100 | 245.6 ±59 | 308.6 ±56 | 0.161 | 736.7 ±105 | 808.1 ±104 | 870.9 ±92 | 923.1 ±86 | 0.959 |
| TGF-α | 1.16 ±0.2 | 0.92 ±0.2 | 1.53 ±0.4 | 1.87 ±0.5 | **0.028*↓** | 1.15 ±0.3 | 1.08 ±0.3 | 0.85 ±0.1 | 1 ±0.2 | 0.318 |
| Fit-3l | 2.54 ±0.3 | 2.75 ±0.4 | 3.52 ±0.5 | 3.23 ±0.6 | 0.574 | 44.51 ±7.6 | 40.26 ±7.8 | 43.52 ±3.8 | 38.61 ±3 | 0.878 |
| PDGF-aa | 6.2 ±1 | 6.93 ±4 | 3.28 ±0.8 | 2.6 ±0.3 | 0.505 | 10.77 ±4 | 11.5 ±4 | 6.47 ±0.7 | 7.74 ±0.7 | 0.721 |
| Pdgf-bb | 79.65 ±15 | 97.13 ±49 | 49.92 ±16 | 36.12 ±5.9 | 0.645 | 69.49 ±26 | 75.31 ±29 | 41.08±12 | 49.91 ±9 | 0.442 |
| Vegf-a | 6 ±1.2 | 4.8 ±0.6 | 5.51 ±0.9 | 5.37 ±0.5 | 0.442 | 2.66 ±0.4 | 2 ±0.3 | 1.77 ±0.3 | 1.6 ±0.4 | 0.645 |
| Scf | 1.49 ±0.4 | 1.47 ±0.4 | 1.04 ±0.4 | 1.04 ±0.4 | 0.833 | 1.72 ±0.4 | 2.08 ±0.4 | 2.04 ±0.3 | 1.83 ±0.2 | **0.046*↑** |
| other | | | | | | | | | | |
| IL-1α | 19.35 ±8.1 | 12.39 ±4.5 | 21.8 ±13.9 | 11.29 ±3.7 | 0.798 | 58.39 ±40.2 | 101.77 ±42.2 | 101.58 ±82 | 114.76 ±83.8 | 0.065 |
| IL-10 | 0.39 ±0.1 | 0.39 ±0.1 | 0.52 ±0.1 | 0.82 ±0.2 | 0.281 | 0.88 ±0.2 | 0.6 ±0.1 | 0.55 ±0.06 | 0.58 ±0.09 | **0.042*↓** |
| IL-15 | 0.79 ±0.1 | 0.82 ±0.1 | 0.91 ±0.1 | 1.17 ±0.2 | 0.798 | 4.09 ±0.9 | 3.13 ±0.8 | 3.11 ±0.4 | 3.19 ±0.3 | 0.161 |
| IL-16 | 101.65 ±30 | 172 ±89 | 127.46 ±40 | 147.25 ±48 | 0.798 | 376.15 ±89 | 336.44 ±56 | 246.6 ±30 | 272.6 ±52 | 0.959 |
| IL-20 | 34.63 ±5 | 31.15 ±5 | 37.63 ±6.6 | 34.63 ±2.4 | 0.345 | 31.41 ±1.9 | 29.63 ±3.7 | 28.5 ±3.5 | 29.65 ±3.8 | 0.874 |
